# Assessment of short-term effects of thoracic radiotherapy on the cardiovascular parasympathetic and sympathetic nervous systems

**DOI:** 10.3389/fnins.2023.1256067

**Published:** 2023-09-04

**Authors:** Shuang Wu, Weizheng Guan, Huan Zhao, Guangqiao Li, Yufu Zhou, Bo Shi, Xiaochun Zhang

**Affiliations:** ^1^School of Medicine, Yangzhou University, Yangzhou, China; ^2^Department of Radiation Oncology, First Affiliated Hospital, Bengbu Medical College, Bengbu, China; ^3^School of Medical Imaging, Bengbu Medical College, Bengbu, China; ^4^Anhui Key Laboratory of Computational Medicine and Intelligent Health, Bengbu Medical College, Bengbu, China; ^5^Department of Oncology, Yangzhou Hospital of Traditional Chinese Medicine, Yangzhou, China

**Keywords:** autonomic modulation, cardiovascular toxicity, deceleration/acceleration capacities of heart rate, heart rate variability, thoracic radiotherapy

## Abstract

**Background:**

Prior research suggests that cardiovascular autonomic dysfunction might be an early marker of cardiotoxicity induced by antitumor treatment and act as an early predictor of cardiovascular disease-related morbidity and mortality. The impact of thoracic radiotherapy on the parasympathetic and sympathetic nervous systems, however, remains unclear. Therefore, this study aimed to evaluate the short-term effects of thoracic radiotherapy on the autonomic nervous system, using deceleration capacity (DC), acceleration capacity (AC) of heart rate, and heart rate variability (HRV) as assessment tools.

**Methods:**

A 5 min electrocardiogram was collected from 58 thoracic cancer patients before and after thoracic radiotherapy for DC, AC, and HRV analysis. HRV parameters employed included the standard deviation of the normal-normal interval (SDNN), root mean square of successive interval differences (RMSSD), low frequency power (LF), high frequency power (HF), total power (TP), and the LF to HF ratio. Some patients also received systemic therapies alongside radiotherapy; thus, patients were subdivided into a radiotherapy-only group (28 cases) and a combined radiotherapy and systemic therapies group (30 cases) for additional subgroup analysis.

**Results:**

Thoracic radiotherapy resulted in a significant reduction in DC (8.5 [5.0, 14.2] vs. 5.3 [3.5, 9.4], *p* = 0.002) and HRV parameters SDNN (9.9 [7.03, 16.0] vs. 8.2 [6.0, 12.4], *p* = 0.003), RMSSD (9.9 [6.9, 17.5] vs. 7.7 [4.8, 14.3], *p* = 0.009), LF (29 [10, 135] vs. 24 [15, 50], *p* = 0.005), HF (35 [12, 101] vs. 16 [9, 46], *p* = 0.002), TP (74 [41, 273] vs. 50 [33, 118], *p* < 0.001), and a significant increase in AC (−8.2 [−14.8, −4.9] vs. -5.8 [−10.1, −3.3], *p* = 0.003) and mean heart rate (79.8 ± 12.6 vs. 83.9 ± 13.6, *p* = 0.010). Subgroup analysis indicated similar trends in mean heart rate, DC, AC, and HRV parameters (SDNN, RMSSD, LF, HF, TP) in both the radiotherapy group and the combined treatment group post-radiotherapy. No statistically significant difference was noted in the changes observed in DC, AC, and HRV between the two groups pre- and post-radiotherapy.

**Conclusion:**

Thoracic radiotherapy may induce cardiovascular autonomic dysfunction by reducing parasympathetic activity and enhancing sympathetic activity. Importantly, the study found that the concurrent use of systemic therapies did not significantly amplify or contribute to the alterations in autonomic function in the short-term following thoracic radiotherapy. DC, AC and HRV are promising and feasible biomarkers for evaluating autonomic dysfunction caused by thoracic radiotherapy.

## Introduction

Thoracic radiotherapy serves as a pivotal element in the treatment of thoracic cancer (TC), including breast cancer, esophageal cancer, lung cancer, and other thoracic malignancies (Raghunathan et al., 2017). Existing research has indicated that thoracic radiotherapy can precipitate vascular endothelial dysfunction, leading to accelerated atherosclerosis and inflammatory activation (Sylvester et al., 2018; Venkatesulu et al., 2018; Baselet et al., 2019). Numerous studies have investigated the amplified risk resulting from thoracic radiotherapy, implicating myocardial, coronary, valvular, and pericardial diseases, along with arrhythmias, as factors contributing to increased mortality (Jacob et al., 2016; Armanious et al., 2018). In this context, the cardiovascular autonomic nervous system (ANS)—a key regulator of heart rate, myocardial function, and myocardial blood flow—assumes significant importance. The initiation of cardiovascular toxicity, as well as early indications of diastolic/systolic dysfunction and disease severity, may stem from cardiovascular autonomic dysfunction (AD) caused by radiation and chemotherapy (Tjeerdsma et al., 1999; Guimarães et al., 2015; Teng et al., 2021). Therefore, examining the impact of thoracic radiotherapy on ANS could provide substantial insights into both short and long-term cardiovascular adversities associated with radiotherapy.

The cardiovascular ANS is constituted by the sympathetic nervous system (SNS) and the parasympathetic nervous system (PNS) (Schwartz and De Ferrari, 2011). ANS functionality can be assessed through several clinically viable measures, such as deceleration capacity (DC), acceleration capacity (AC) of heart rate, and heart rate variability (HRV) (Bauer et al., 2006a; Lombardi and Stein, 2011; Zou et al., 2016). Previous research has underscored the prognostic value of DC, AC, and HRV in the early detection of cardiovascular disease onset and sudden cardiac death rates in patients with conditions like myocardial infarction, heart failure, dilated cardiomyopathy, or coronary artery disease (Arsenos et al., 2016; Zou et al., 2016; Wang et al., 2017; Rizas et al., 2018; Fang et al., 2020). More recent observational studies have established that DC is a potent predictor of cardiovascular toxicity stemming from treatments like epirubicin or trastuzumab in breast cancer patients (Feng and Yang, 2015; Feng et al., 2021). Hence, the incorporation of DC, AC, and HRV in clinical studies investigating potential cardiovascular toxicity resulting from antitumor therapy could prove beneficial.

Earlier studies have provided preliminary insights into the effects of antitumor treatment on cardiovascular ANS in patients with malignant tumors (Ekholm et al., 2000; Groarke et al., 2015; Stachowiak et al., 2018; Caru et al., 2019). Early identification of cardiovascular AD has the potential to enhance preventative strategies to mitigate clinically significant cardiac toxicity subsequent to thoracic radiotherapy. It may be necessary to identify novel biomarkers in order to facilitate the early detection of heart damage resulting from radiation exposure. Therefore, the objective of this study is to elucidate the alterations in DC, AC, and HRV before and after thoracic radiotherapy in TC patients, contributing novel insights into the short-term radiotherapy effects on cardiovascular ANS.

## Materials and methods

### Participants

This study included TC patients who underwent thoracic radiotherapy at the Department of Tumor Radiotherapy, the First Affiliated Hospital of Bengbu Medical College. Exclusion criteria were as follows: (1) presence of a pacemaker, (2) prior receipt of thoracic radiotherapy, (3) incomplete thoracic radiotherapy, and (4) poor electrocardiogram (ECG) quality. The study was approved by the local hospital’s Clinical Medical Research Ethics Board (registration number: 2019KY031). All participants were voluntary contributors who provided informed consent.

Patients were administered thoracic radiotherapy via a linear accelerator (Siemens or Elekta Synergy Platform) using 6-MV photon beams. The median cumulative radiation dose was 50 Gray, while the median individual radiation dose was 2 Gray. Certain patients also received potentially cardiotoxic systemic therapies, such as taxanes, platinum compounds, or trastuzumab, which may instigate variations in the cardiovascular ANS (Coumbe and Groarke, 2018; Teng et al., 2021). Consequently, we partitioned TC patients into two groups: the radiotherapy-only group, and the combined radiotherapy and systemic therapies group.

### Data collection

An ECG was captured before and after radiotherapy using an ECG recorder (HeaLink-R211B; HeaLink Ltd., Bengbu, China). A V6-lead was employed, and ECGs were collected at a sampling rate of 400 Hz. Participants remained in a supine position, motionless for 5 min during ECG collection. The same operator conducted measurements both pre- and post-radiotherapy.

### Deceleration/acceleration capacities of heart rate and heart rate variability analysis

The Pan-Tompkins algorithm was employed in this study to extract the R-peaks from the ECG readings (Pan and Tompkins, 1985). The Kubios software’s threshold-based automatic artifact correction algorithm was used to rectify both technical and physiological artifacts (Niskanen et al., 2004). The mean heart rate (HR) was defined as the average resting heart rate over a period of 5 min. The phase-rectified signal averaging technique was utilized for the computation of the DC and AC. Initially, the R-R intervals time series were examined to detect the decelerating and accelerating anchors that were characterized by a longer or shorter value than the preceding value, respectively. Subsequently, R-R intervals segments surrounding the decelerating and accelerating anchors were evaluated. Lastly, the aforementioned segments were aligned at the decelerating and accelerating anchors, and the signals of segments were averaged to derive the phase-rectified signal averaging signals (Bauer et al., 2006a,b; Nasario-Junior et al., 2014). Several commonly used HRV parameters were engaged in this study: the standard deviation of the normal-normal intervals (SDNN), root mean square of successive interval differences (RMSSD), low frequency power (LF, 0.04–0.15 Hz), high frequency power (HF, 0.15–0.4 Hz), total power (TP, 0–0.4 Hz), and the ratio of LF to HF (LF/HF). For the frequency domain HRV analysis, R-R interval sequences were transformed into evenly sampled time series using a 4 Hz resampling rate, with the aid of a cubic spline interpolation method. The fast Fourier transform algorithm was applied in tandem with Welch’s periodogram method (150 s window width and 50% overlap window) to calculate HRV spectra.

DC is a quantitative metric of the PNS regulatory ability, while AC symbolizes the SNS tone. SDNN represents the overall variability of HRV, reflecting the combined activity of the PNS and SNS. RMSSD is indicative of vagus nerve activity, LF is influenced by both PNS and SNS, and HF corresponds to the PNS tone. TP is representative of the activities of the PNS and SNS, while the LF/HF ratio indicates the interplay between the SNS and PNS (Bauer et al., 2006a; Vanderlei et al., 2009; Lombardi and Stein, 2011; Zou et al., 2016; Thomas et al., 2019).

All DC, AC, and HRV indicators were analyzed using Kubios HRV Premium software (version 3.5, Magi Kubios Oy, Kuopio, Finland^1^) (Niskanen et al., 2004).

### Statistical analysis

The Shapiro–Wilk test was applied to verify the normality of the data. The independent sample *t*-test or Mann–Whitney *U* test was utilized to compare the differences in each continuous variable, and the Chi-square test was employed to compare the differences in each counting variable among the subgroups before thoracic radiotherapy. The differences in DC, AC, and HRV before and after radiotherapy were analyzed by the Paired Sample *t*-test or Wilcoxon sign-rank test. Cohen’s *d* value characterized the effect sizes of the differences in DC, AC, and HRV before and after radiotherapy in the subgroups. The independent sample *t*-test or Mann–Whitney *U* test was used to compare the differences in DC, AC, and HRV before and after radiotherapy among the subgroups. All of these statistical analyses were conducted using SPSS Statistics 25.0 (IBM Corp., Chicago, Illinois, United States of America). All tests were two-tailed, with *p* values of <0.05 considered statistically significant.

## Results

A total of 58 patients diagnosed with TC were considered, consisting of 25 males and 33 females, with an average age of 58.6 ± 12.1 years. The prevalence of specific cancer types varied among the participants, with esophageal cancer being the most common (20/58), followed by breast (19/58), lung (16/58), Hodgkin’s lymphoma (1/58), thymic cancer (1/58), and lung metastasis (1/58). The patients were categorized into two cohorts based on their treatment regimen during thoracic radiotherapy: one received solely radiotherapy (28 patients) while the other received a combination of radiotherapy and systemic therapies (30 patients). The overall patient characteristics and a comparison of variables for subgroups pre-radiotherapy are detailed in Table 1.

**Table 1:** General characteristics of patients with comparisons of variables for subgroups prior to radiotherapy.

Variables	All (*N* = 58)	Radiotherapy group (*N* = 28)	Radiotherapy with systemic therapies group (*N* = 30)	*p*
Gender (Male/Female)	25/33	10/18	15/15	0.272
Age (years)	58.6 ± 12.1	56.1 ± 11.3	60.9 ± 12.6	0.130
BMI (kg/m^2^)	23.3 ± 3.6	23.9 ± 3.3	22.7 ± 3.8	0.188
Hypertension (yes/no)	14/44	6/22	8/22	0.641
Diabetes (yes/no)	5/53	2/26	3/27	1.000
Diagnosis (BC/EC/LC/others)	19/20/16/3	13/5/8/2	6/15/8/1	**0.049**
Mean HR (bpm)	79.8 ± 12.6	82.9 ± 10.6	76.9 ± 13.7	0.070
DC (ms)	8.5 [5.0, 14.2]	7.8 [5.3, 16.8]	9.8 [3.5, 14.2]	0.767
AC (ms)	−8.2 [−14.8, −4.9]	−7.6 [−17.3, −5.1]	−9.0 [−14.8, −4.0]	0.744
SDNN (ms)	9.9 [7.0, 16.0]	9.9 [6.9, 17.7]	9.8 [7.0, 16.0]	0.950
RMSSD (ms)	9.9 [6.9, 17.5]	9.8 [6.6, 15.9]	9.9 [7.2, 18.0]	0.889
LF (ms^2^)	29 [10, 135]	44 [13, 133]	25 [9, 140]	0.455
HF (ms^2^)	35 [12, 101]	34 [13, 119]	44 [10, 101]	0.913
TP (ms^2^)	74 [41, 273]	72 [40, 301]	74 [41, 273]	0.852
LF/HF	0.936 [0.379, 2.618]	0.905 [0.463, 2.506]	1.051 [0.303, 2.948]	0.630

Values are expressed as the number of patients or mean ± standard deviation or median [1st quartile, 3rd quartile].

Bold *p* values indicate statistical significance (*p*-value < 0.05).

N, number of individuals; BMI, body mass index; BC, breast cancer; EC, esophageal cancer; LC, lung cancer.

Our statistical analysis revealed significant variations in mean HR, DC, AC, SDNN, RMSSD, LF, HF, and TP across all patients when data prior to radiotherapy were compared. Specific changes included a significant decrease in DC (*p* = 0.002), SDNN (*p* = 0.003), RMSSD (*p* = 0.009), LF (*p* = 0.005), HF (*p* = 0.002), and TP (*p* < 0.001) when compared with pre-radiotherapy data. Contrastingly, AC (*p* = 0.003) and mean HR (*p* = 0.010) increased post-radiotherapy. A higher LF/HF ratio was observed post-radiotherapy, although this difference was not statistically significant (Table 2).

**Table 2:** Comparison of variables for all patients before and after radiotherapy.

Variables	Pre-values	Post-values	*p*
Mean HR (bpm)	79.8 ± 12.6	83.9 ± 13.6	**0.010**
DC (ms)	8.5 [5.0, 14.2]	5.3 [3.5, 9.4]	**0.002**
AC (ms)	−8.2 [−14.8, −4.9]	−5.8 [−10.1, −3.3]	**0.003**
Time-domain indices of HRV			
SDNN (ms)	9.9 [7.03, 16.0]	8.2 [6.0, 12.4]	**0.003**
RMSSD (ms)	9.9 [6.9, 17.5]	7.7 [4.8, 14.3]	**0.009**
Frequency-domain indices of HRV			
LF (ms^2^)	29 [10, 135]	24 [15, 50]	**0.005**
HF (ms^2^)	35 [12, 101]	16 [9, 46]	**0.002**
TP (ms^2^)	74 [41, 273]	50 [33, 118]	**< 0.001**
LF/HF	0.936 [0.379, 2.618]	1.281 [0.631, 2.395]	0.559

Values are expressed as mean ± standard deviation or median [1st quartile, 3rd quartile].

Bold *p* values indicate statistical significance (*p*-value < 0.05).

**Figure 1:**
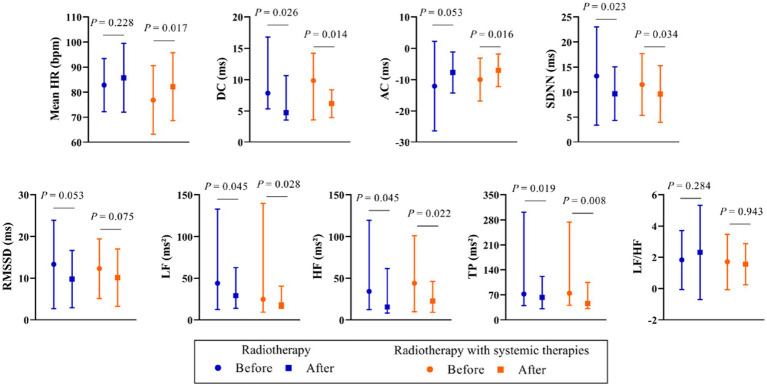
Differences in parameters for subgroups of patients pre- and post-radiotherapy.

**Figure 2:**
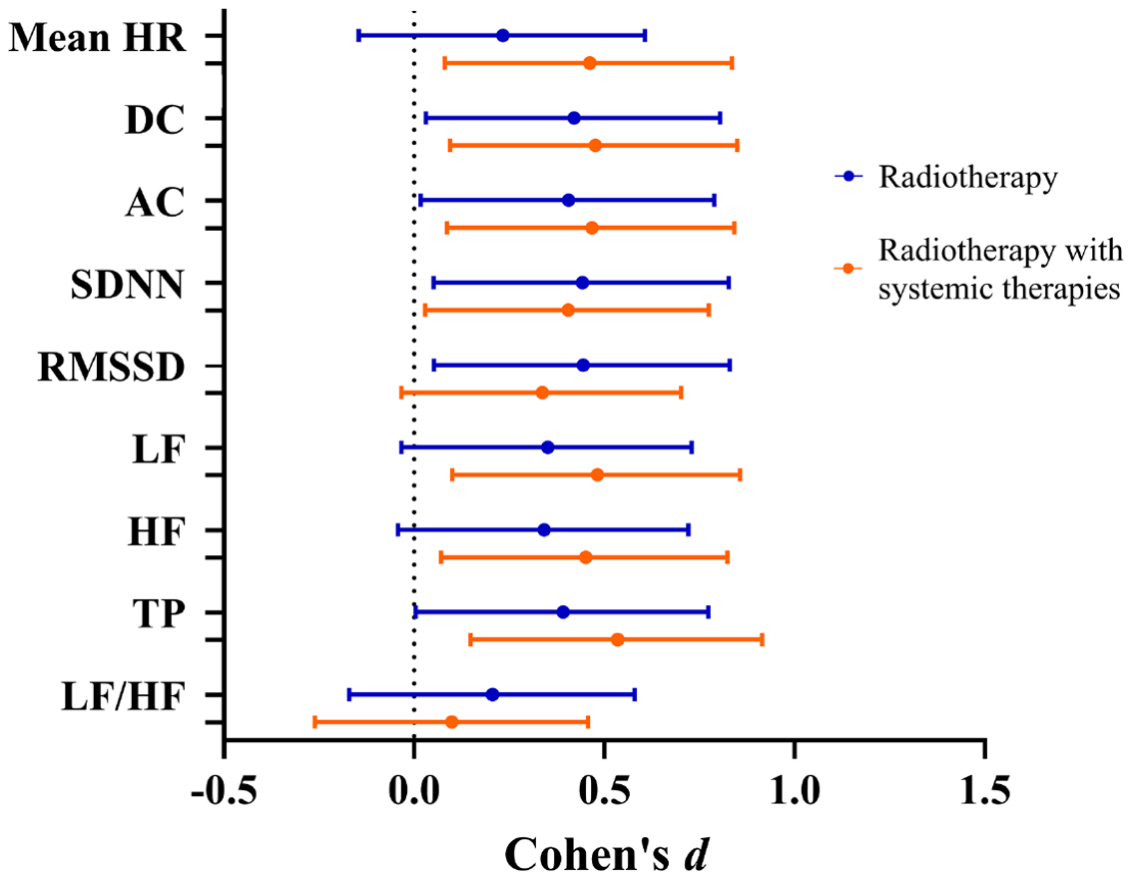
Effect size of variables for subgroups pre- and post-radiotherapy.

The subgroup analysis demonstrated that mean HR, DC, AC, as well as HRV parameters SDNN, RMSSD, LF, HF, and TP followed a consistent trend of increase or decrease post-thoracic radiotherapy in both the radiotherapy and combined therapy groups (Figure 1). The effect sizes of DC, AC, and HRV within subgroups are illustrated in Figure 2.

**Figure 3:**
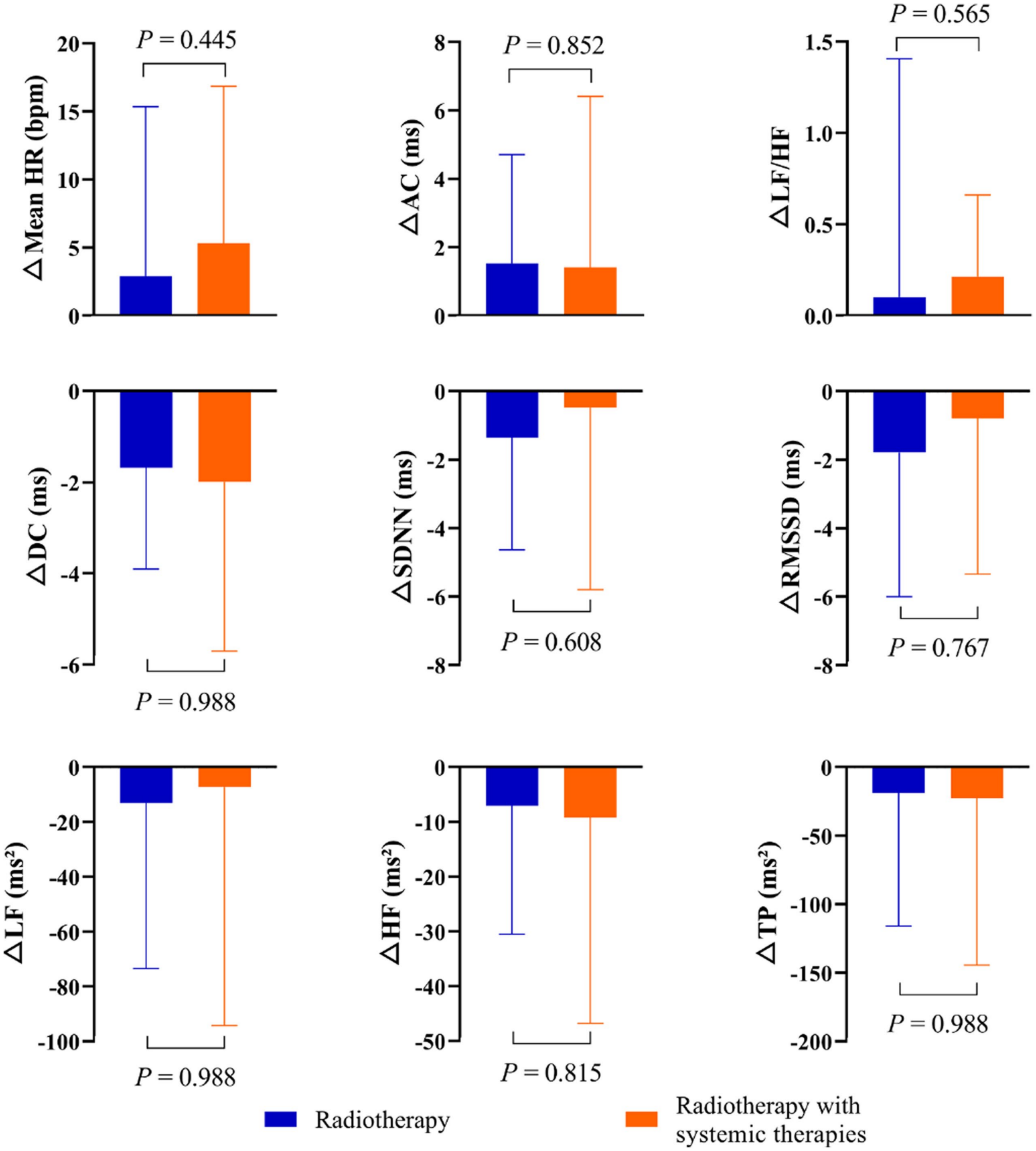
Change in DC, AC, and HRV, calculated as post-radiotherapy value minus pre-radiotherapy value for subgroups of patients.

To examine whether the short-term effects of combined thoracic radiotherapy and systemic therapies on the cardiovascular ANS were either synergistic or additive, we analyzed the variations in DC, AC, and HRV before and after radiotherapy for subgroup patients. However, no statistically significant differences were found in the changes of DC, AC, and HRV between the two groups pre- and post-radiotherapy (Figure 3).

## Discussion

This study evaluated the immediate effects of thoracic radiotherapy on DC, AC, and HRV in TC patients. Our findings demonstrated that thoracic radiotherapy could lead to significant alterations in mean HR, DC, AC, and HRV parameters including SDNN, RMSSD, LF, HF, and TP. Additionally, a subgroup analysis was performed, taking into account patients who were also receiving systemic therapies concurrently with radiotherapy. The outcomes implied that mean HR, DC, AC, SDNN, RMSSD, LF, HF, and TP demonstrated identical trends of increase or decrease post-radiotherapy, in both the radiotherapy-only group and the combined radiotherapy and systemic therapies group. Importantly, there was no substantial difference observed in the alterations of DC, AC, and HRV between the two groups before and after the radiotherapy.

The pathophysiology of cardiovascular AD induced by thoracic radiotherapy and systemic therapies is multifaceted and complex. The primary mechanisms underlying thoracic radiotherapy-associated AD encompass direct neural damage caused by radiation and the pro-inflammatory state inherent in malignancies (Teng et al., 2021). The direct radiation exposure to the vagal nerve and carotid regions may instigate inflammation and subsequent fibrosis, leading to potential damage to the vagal nerve and carotid sinus baroreflexes (Sharabi et al., 2003; Goodman and Schrader, 2009; Kong et al., 2011). An alternate explanation for the incidence of cardiovascular AD in cancer survivors could be the modification of the ANS as a consequence of a pro-inflammatory state, as opposed to intrinsic neural damage. This inflammatory state is frequently observed in cancer settings, particularly at the initiation of radiotherapy, triggering an overproduction of inflammatory cytokines such as interleukin-1, interleukin-6, and tumor necrosis factor-alpha. These factors may suppress vagal nerve activity, which in turn results in an elevation of the resting heart rate and a shift in the balance between the PNS and SNS toward SNS predominance (Thayer and Lane, 2007). Systemic therapies, such as chemotherapy or targeted therapy, may also affect the ANS during and after cancer treatment. The etiology of chemotherapy-induced AD, involving agents like anthracyclines, taxanes, and platinum compounds, may involve inflammatory pathways, cellular injury, and oxidative stress (Coumbe and Groarke, 2018; Teng et al., 2021).

The stability of heart rhythm is primarily determined by the combined effects of the PNS and SNS (Lahiri et al., 2008). Recent research demonstrated that patients with Hodgkin’s lymphoma who underwent thoracic radiotherapy presented a higher mean HR in contrast to those who did not undergo such treatment (Groarke et al., 2015). Moreover, previous findings strongly correlated an elevated mean HR with increased morbidity and mortality rates of cardiovascular diseases (Cooney et al., 2010; Anker et al., 2016; Lee et al., 2016). In this study, thoracic radiotherapy was found to increase the mean HR of all TC patients. A subgroup analysis revealed that patients who underwent radiotherapy demonstrated a higher mean HR post-treatment, although the difference was not statistically significant. This observation suggests that an elevated mean HR could be an efficient, albeit not entirely accurate, indicator for a rapid assessment of cardiovascular AD.

Numerous studies have emphasized the close correlation between neck or chest irradiation and cardiovascular AD (Hoca et al., 2012; Huang et al., 2013; Goyal et al., 2017). For instance, an investigation involving 14 TC patients discovered that mediastinal radiotherapy resulted in a decrease in HF values and an increase in the LF/HF in HRV frequency domain parameters (Hoca et al., 2012). Our research corroborated that thoracic radiotherapy negatively impacts HRV indicators, including SDNN, RMSSD, LF, HF, and TP. These findings suggest that thoracic radiotherapy may contribute to AD by diminishing PNS activity. Nevertheless, our study found no statistically significant difference in LF/HF values pre- and post-radiotherapy, which might be attributable to the complex physiological basis of LF/HF. Pagani et al. (1984) proposed the use of LF/HF to measure cardiac sympatho-vagal balance. However, several studies have contested this approach, asserting that LF/HF fails to accurately quantify the dynamic relationship between SNS and PNS activities and cannot confidently depict the physiological basis of LF/HF (Billman, 2011; Billman, 2013). DC and AC represent emerging noninvasive techniques for evaluating autonomic modulation (Bauer et al., 2006a; Zou et al., 2016). Recent observational studies have shown that DC was an effective predictor of epirubicin-related or trastuzumab-related cardiotoxicity development in patients with breast cancer (Feng and Yang, 2015; Feng et al., 2021). Our study determined that thoracic radiotherapy can significantly decrease DC while simultaneously increasing AC. This indicates that thoracic radiotherapy may lead to a reduction in PNS tone, as reflected by DC, and an increase in SNS activity, as assessed by AC, in TC patients. The changes in DC and AC precipitated by thoracic radiotherapy may correlate with the incidence of cardiovascular disease and sudden cardiac death rates in TC patients post-radiotherapy. However, these potential relationships warrant verification through long-term follow-up studies.

Prior research has established the potential cardiovascular toxicity of taxanes, platinum compounds, anthracyclines, and trastuzumab (Jain et al., 2017; Coumbe and Groarke, 2018; Dong and Chen, 2018). For instance, Dermitzakis et al. (2016) found that concurrent paclitaxel and carboplatin chemotherapy significantly influenced the PNS and SNS in ovarian cancer patients, predominantly affecting parasympathetic heart innervation. Liu et al. (2023) showed that the combination of taxane and carboplatin chemotherapy could impact early ANS status in patients with cervical cancer. Meinardi et al. (2001) discovered that even moderate doses of epirubicin were not associated with persistent alterations in HRV for breast cancer patients, suggesting a possible dose–response relationship for anthracycline-induced ANS damage. Our findings revealed no statistically significant difference in the alterations of DC, AC, and HRV pre- and post-radiotherapy between the radiotherapy-only group and the combined radiotherapy and systemic therapies group. This implies that, in the short term, thoracic radiotherapy coupled with systemic therapies does not significantly exacerbate or add to ANS changes. Currently, while radiotherapy and systemic therapies may exert cardiac toxic effects, the precise mechanism of their interaction remains elusive.

Emerging evidence has shown that patients receiving thoracic radiotherapy have an increased risk for developing cardiovascular disease (Groarke et al., 2015; Jacob et al., 2016; Armanious et al., 2018). According to the American Society of Clinical Oncology’s clinical practice guideline, baseline assessment of the left ventricular ejection fraction could feasibly stratify risk in patients potentially susceptible to cardiovascular toxicity (Armenian et al., 2017). However, relying solely on left ventricular ejection fraction as an indicator of cardiac performance has faced substantial criticism (Konstam and Abboud, 2017; Marwick, 2018). Prior research has demonstrated that cardiovascular ANS function may be a more accurate mortality predictor following myocardial infarction than left ventricular ejection fraction (Bauer et al., 2006a). Additional studies have emphasized the significance of alterations in serum biomarkers, notably cardiac troponin, for forecasting cardiovascular toxicity (Cardinale et al., 2010; Pavo et al., 2015). Therefore, we propose a baseline assessment encompassing ANS function, left ventricular ejection fraction, and troponin measurement for cardiovascular toxicity risk stratification, with subsequent combined measurements for longitudinal cardiac monitoring.

## Limitations

Our study’s primary limitation is the heterogeneity of the study population. Given our limited sample size, it was unattainable to discern the distinct performance of DC, AC, and HRV in patients with varying types of TC. We strongly advocate for future studies to augment the sample size and prioritize these differences (e.g., tumor type, chemotherapy drugs and doses). Furthermore, due to limited statistical power, some background variables potentially influencing cardiovascular ANS, such as physical activity, were omitted.

## Conclusion

Our study elucidates the modifications of DC, AC, and HRV pre- and post-thoracic radiotherapy in patients diagnosed with TC. These results suggest that thoracic radiotherapy prompts cardiovascular AD by diminishing PNS activity and augmenting SNS tone. Additionally, the data implies that combining radiotherapy with systemic therapies may not yield a substantial synergetic or additive impact on the ANS in the short term. We hypothesize that the simultaneous measurement of DC, AC, and HRV could be helpful in developing an integrated biomarker for identifying both short- and long-term radiotherapy-induced cardiac autonomic modulation impairments.

## Data availability statement

The raw data supporting the conclusions of this article will be made available by the authors, without undue reservation.

## Ethics statement

The studies involving humans were approved by the Medical Ethics Committee of the First Affiliated Hospital of Bengbu Medical College. The studies were conducted in accordance with the local legislation and institutional requirements. The participants provided their written informed consent to participate in this study.

## Author contributions

SW: Data curation, Writing – original draft. WG: Formal analysis, Writing – original draft. HZ: Writing – original draft. GL: Writing – original draft. YZ: Resources, Supervision, Writing – review & editing. BS: Conceptualization, Methodology, Resources, Writing – review & editing, XZ: Resources, Supervision, Writing – review & editing.

## Funding

This research was funded by the “512” Outstanding Talents Fostering Project of Bengbu Medical College (grant number BY51201312), the Natural Science Research Project of Anhui Educational Committee (grant number KJ2021A0803), and the Scientific Research Innovation Project of Bengbu Medical College (grant number BYKC201905).

## Conflict of interest

An immediate family member of BS owns stock in HeaLink Ltd., Bengbu, China.

The remaining authors declare that the research was conducted in the absence of any commercial or financial relationships that could be construed as a potential conflict of interest.

## Publisher’s note

All claims expressed in this article are solely those of the authors and do not necessarily represent those of their affiliated organizations, or those of the publisher, the editors and the reviewers. Any product that may be evaluated in this article, or claim that may be made by its manufacturer, is not guaranteed or endorsed by the publisher.

## Footnotes

^1^ https://www.kubios.com
